# Chitosan promotes cancer progression and stem cell properties in association with Wnt signaling in colon and hepatocellular carcinoma cells

**DOI:** 10.1038/srep45751

**Published:** 2017-04-03

**Authors:** Po-Hsiang Chang, Keisuke Sekine, Hsiao-Mei Chao, Shan-hui Hsu, Edward Chern

**Affiliations:** 1 niChe Lab for Stem Cell and Regenerative Medicine, Department of Biochemical Science and Technology, National Taiwan University, Taipei 10617, Taiwan; 2 Department of Regenerative Medicine, Yokohama City University Graduate School of Medicine, Kanagawa 236-0004, Japan; 3 Department of Pathology, Wan Fang Hospital, Taipei Medical University, Taipei 11696, Taiwan; 4 Institute of Polymer Science and Engineering, National Taiwan University, Taipei 10617, Taiwan

## Abstract

Cancer stem cells (CSCs), a small population of cancer cells, have been considered to be the origin of cancer initiation, recurrence, and metastasis. Tumor microenvironment provides crucial signals for CSCs to maintain stem cell properties and promotes tumorigenesis. Therefore, establishment of an appropriate cell culture system to mimic the microenvironment for CSC studies is an important issue. In this study, we grew colon and hepatocellular carcinoma (HCC) cells on chitosan membranes and evaluated the tumor progression and the CSC properties. Experimental results showed that culturing cancer cells on chitosan increased cell motility, drug resistance, quiescent population, self-renewal capacity, and the expression levels of stemness and CSC marker genes, such as OCT4, NANOG, CD133, CD44, and EpCAM. Furthermore, we demonstrated that chitosan might activate canonical Wnt/β-catenin-CD44 axis signaling in CD44^positive^ colon cancer cells and noncanonical Wnt-STAT3 signaling in CD44^negative^ HCC cells. In conclusion, chitosan as culture substrates activated the essential signaling of CSCs and promoted CSC properties. The chitosan culture system provides a convenient platform for the research of CSC biology and screening of anticancer drugs.

Cancer is one of the major leading causes of death worldwide and associated with mortality and morbidity^1^. The common anticancer therapies such as radiotherapy and chemotherapy may lead to drug resistance and further subsequent cancer recurrence or metastasis. Emerging evidences indicate that certain subpopulations of cancer cells in a tumor could be the origin of the tumor. They share some similar properties with stem cells and are named as cancer stem cells (CSCs)^2^,^3^. These cells possess higher migration ability that is associated with invasion and metastasis^4^. They also stay at a slow-cycling/quiescent state to resist anti-proliferation drugs^5^. CSCs express specific surface markers such as CD133, EpCAM, and CD44 that are used for CSC identification and isolation^6^. CSCs can self-renew to maintain CSC pools and differentiate into heterogeneous progeny cancer cells^7^. Signaling cascades within CSCs such as Notch, STAT3, and Wnt/β-catenin are dysregulated to maintain their stem cell properties^8^. In colon cancers, Wnt/β-catenin is essential to maintain the CSC population. Stimulation of the Wnt/β-catenin signaling on differentiated colon cancer cells can restore CSC properties^9^. Noncanonical Wnt5-Frizzled2 pathway also regulates epithelial-mesenchymal transition (EMT), a characteristic of CSCs, and promotes metastasis in hepatocellular carcinoma (HCC) and colon cancer cells through the activation of STAT3^10^. The phosphorylated STAT3 is observed in CSCs to upregulate the stemness properties^11^. Targeting CSCs and the specific essential signaling can provide novel therapeutic strategies^12^. However, due to the scarcity of CSCs within the tumors^2^, enrichment of CSCs *in vitro* is crucial for studies of CSC biology and applications in drug screening.

In tumor microenvironments, extracellular matrix (ECM) and stromal cells support cancer development and stemness^13^. Recent studies showed that some biocompatible materials may mimic tumor-associated ECM^14^. A few groups have used the biomaterials to create three-dimension (3D) scaffolds to culture cancer cells^15^,^16^. For example, ovarian cancer cells embedded within gelatin-methacrylamide hydrogels displayed a higher drug resistance^17^. Ewing sarcoma cells in porous electrospun polycaprolactone scaffolds exhibited the expression signaling patterns similar to tumors *in vivo*^18^. Besides, previous studies revealed that crosstalks between CSCs and the microenvironment could maintain the properties of CSCs^19^,^20^. Chang *et al*. showed that the laminin 511 matrix, one type of ECM, supported and maintained the breast CSC properties^21^. Therefore, establishment of a simple biomaterial platform to culture cancer cells is an essential step to investigate cancer stemness.

Chitosan is a cationic natural polysaccharide derived from chitin and is applied in many biomedical fields because of its antibacterial activity, biocompatibility, and biodegradability^22^. Chitosan crosslinked with other molecules has also been used to culture cancer cells^23^,^24^. For instance, porous chitosan-alginate scaffolds may enrich the CD133^+^ glioblastoma CSC population^25^ and electrospun polycaprolactone-chitosan scaffolds may increase the breast cancer stemness^26^. However, the mechanisms of interactions between cells and biomaterials are still unknown. Our previous study showed that chitosan (CS) membranes and hyaluronan (HA) grafted chitosan (CSHA) membranes could increase the stemness of mesenchymal stem cells (MSCs)^27^. HA is the principal ligand of receptor CD44 that is aberrantly expressed on the surface of CSCs^28^. CSHA membranes promoted the aggressiveness of lung cancer cells possibly via the interaction of HA with CD44 receptor^29^. Yet, the influence of chitosan itself on cancer cells remains to be elucidated.

Rao *et al*. recently demonstrated that chitosan nanoparticles could bind CSCs via CD44 receptor, a major target gene of Wnt signaling^30^,^31^. This finding implied that the chemical properties of chitosan might resemble HA to some extent. Here, we attempted to clarify the effect of chitosan without any surface modification on colon cancer cells and HCC cells. We showed that chitosan itself promoted CSC-related characteristics and tumor progression of not only CD44^positive^ colon cancer cells but also CD44^negative^ HCC cells. We unraveled the mechanisms regarding how chitosan substrates promoted cancer stemness and revealed that canonical and noncanonical Wnt signaling pathways played key roles in the interactions between chitosan and cancer cells. Chitosan as culture substrates may provide a convenient platform that mimics ECM for the research of CSC biology and drug screening.

## Results

### Morphologies of colon cancer and HCC cell lines on CS and CSHA membranes

**Figure 1:**
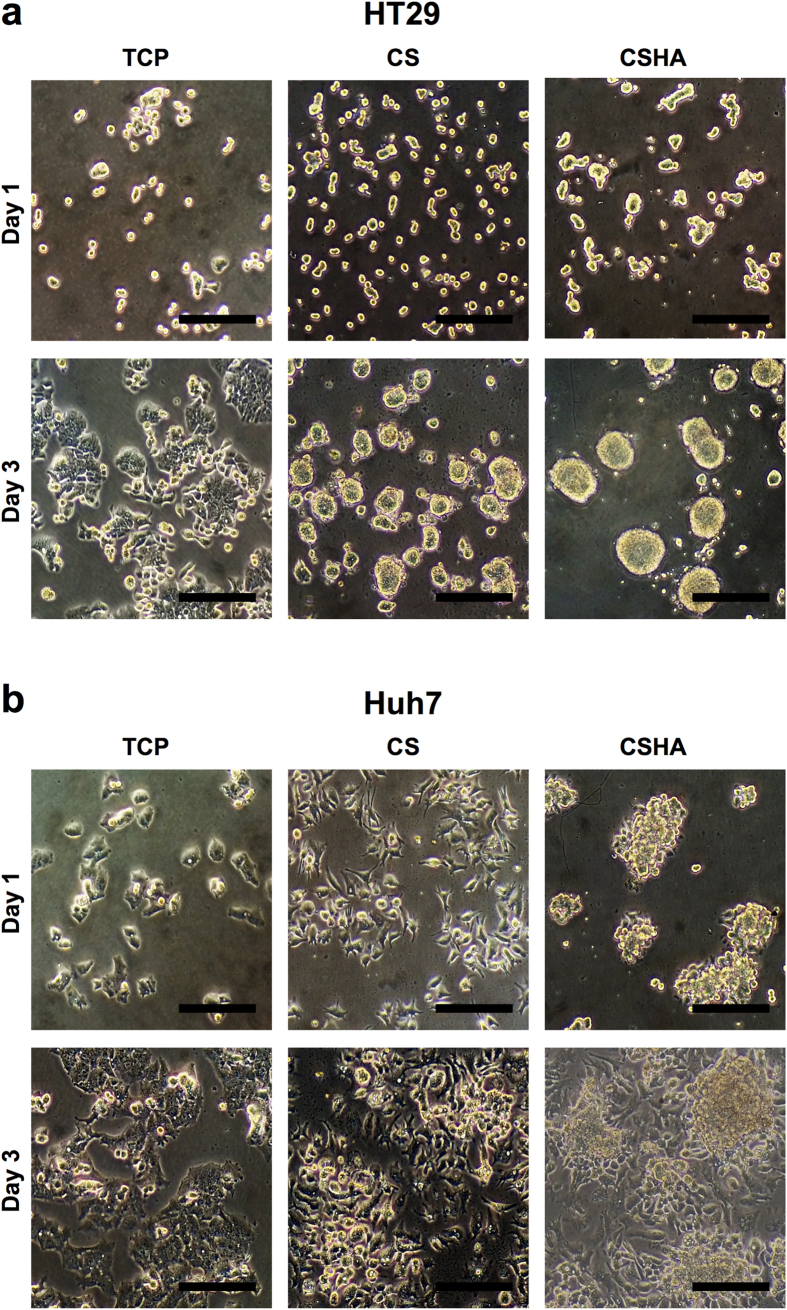
Morphology of cancer cells on CS and CSHA membranes. (**a**) Colon cancer cell line HT29 cultured for 24 and 72 hr. (**b**) HCC cell line Huh7 cultured for 24 and 72 hrs. Scale bar represents 200 μm.

To investigate how CS and CSHA membranes directly affect the phenotypes of colon cancer cell lines HT29, DLD-1, HCT116, SW480 and HCC cell lines Huh7, HepG2, Hep3B, SKHep-1, we cultured the cells on membranes for 72 hrs following observation per 24 hrs. All cell lines attached on tissue culture polystyrene (TCP) plates and demonstrated the normal epithelial phenotype, but cells cultured on CS and CSHA membranes displayed different morphologies. Among colon cancer cell lines, only HT29 formed spheroids on both CS and CSHA membranes (Fig. 1a, Supplementary Fig. S1, Supplementary video). For HCC cell lines, most of them remained epithelial phenotypes on CS membranes but formed clusters on CSHA membranes (Fig. 1b, Supplementary Fig. S1, Supplementary video). Meanwhile, Huh7 aggregated into colonies on both CS and CSHA membranes.

### CS promoted cell motility and drug resistance

**Figure 2:**
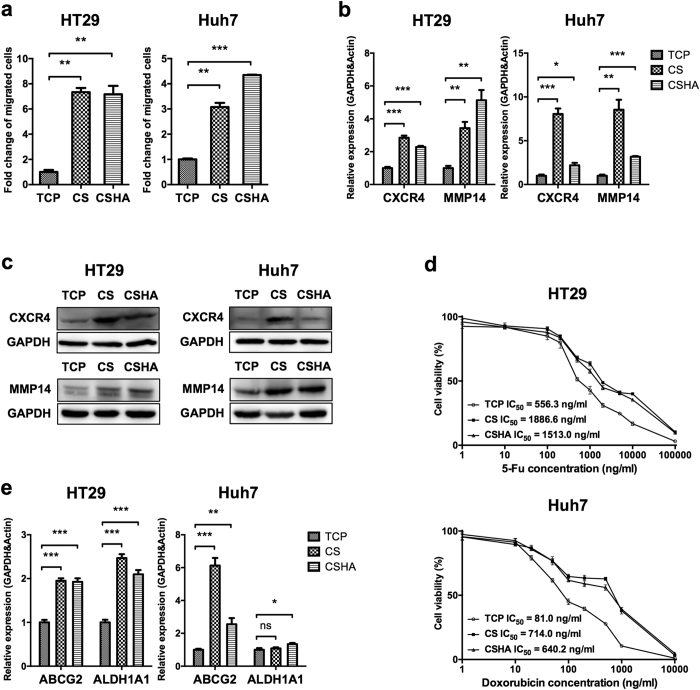
Analysis of cell motility and drug resistance and associated gene expressions. (**a**) The migrated cell numbers were quantified by the transwell migration assay and normalized to the TCP group. (**b**) CXCR4 and MMP14 mRNA expressions after 72 hrs of culture. (**c**) The protein expression level of CXCR4 and MMP14 after 72 hrs of culture. (**d**) Cell viability after treatments with varying concentrations of 5-Fu (1, 10, 100, 200, 500, 1,000, 2,000, 5,000, 10,000, and 100,000 ng/mL) and doxorubicin (1, 10, 20, 50, 100, 200, 500, 1,000, and 10,000 ng/mL) for HT29 and Huh7, repectively. Each point represents the means of six determinations (n = 6). (**e**) The mRNA expression levels of ABCG2 and ALDH1A1 after 72 hrs of culture. Each bar represents the means of three determinations ± SD. *p < 0.05, **p < 0.01, ***p < 0.001 among the indicated groups.

The *in vitro* transwell assay was used to examine the effect of CS and CSHA substrates on cell migration. As shown in Fig. 2a, the migration ability of both HT29 and Huh7 was promoted on either CS or CSHA membranes. In addition, cells cultured on CS and CSHA membranes increased the expression of CXCR4 and MMP14 in both HT29 and Huh7 (Fig. 2b,c). Moreover, knockdown of MMP14 reduced the migration ability of both cell lines, whereas knockdown of CXCR4 only reduced the migration ability of HT29 (Supplementary Fig. S2). To understand whether cells cultured on CS and CSHA membranes increased the drug resistance, we employed two different chemotherapeutic drugs, 5-Fluorouracil and doxorubicin, to treat HT29 and Huh7 respectively. The results revealed that cells cultured on CS and CSHA membranes had higher viability than those cultured on TCP plates (Fig. 2d). Upon drug treatment, the IC_50_ values for HT29 and Huh7 grown on TCP plates were 556.3 and 81.0 ng/mL for 5-Fu and doxorubicin, respectively. The values of IC_50_ increased to 1886.6 and 714.0 ng/mL for HT29 and Huh7 on CS membranes. Similarly, the values of IC_50_ increased to 1513.0 and 640.2 ng/mL for those cells on CSHA membranes. Moreover, the expression level and enzyme activity of ALDH1A1 increased for HT29 and Huh7 cultured on CS and CSHA membranes (Fig. 2e, Supplementary Fig. S3). On the other hand, the expression level and function of ABCG2 significantly increased for both HT29 and Huh7 on CS and CSHA membranes (Fig. 2e, Supplementary Fig. S4).

### Increased stemness and cancer properties analyzed by microarray gene expression

**Figure 3:**
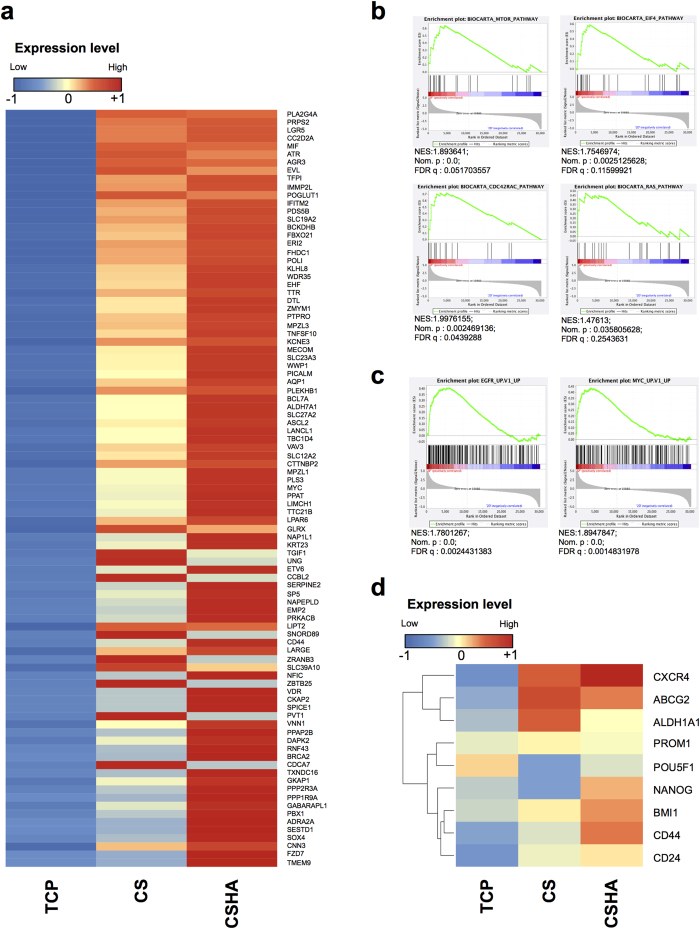
Microarray analysis of colon cancer cell line HT29 grown on TCP plates, CS, and CSHA membranes. (**a**) Heatmap of expression levels for genes related with colon stem cells. (GEO: GSE93704) (**b**) GSEA anaysis of gene set related to mTOR, elF4, Cdc42, and Ras signaling pathways. (**c**) GSEA anaysis of EGFR and Myc target gene set. Normalized Enrichment Score (NES), Nominal p-value (NOM.p) and FDR q-value (FDRq) were shown in the figure. (**d**) Heatmap of expression levels for genes associated with cancer stemness and CSC properties such as migration and drug resistance.

Transcriptome analysis was performed on the colon cancer cell line HT29 harvested from various substrates. Compared to TCP plates, cells cultured on CS or CSHA membranes increased the expression of intestinal stem cell related genes (Fig. 3a). The results of Gene Set Enrichment Analysis (GSEA) indicated that cells on chitosan substrates increased the genes related to mTOR, eIF4, Cdc42, and Ras signaling pathways, which are associated to the cancer progression and invasiveness (Fig. 3b). CS substrates also promoted the expressions of EGFR/Myc target genes that modulate cancer proliferation (Fig. 3c). Besides, cancer stemness core genes were also upregulated in the cells cultured on the CS membranes (Fig. 3d). These transcriptome analysis data indicated that the cultivated cells on CS substrates increased the expression of genes related to stemness and cancer progression.

### CS increased the stemness and the population of CSCs

**Figure 4:**
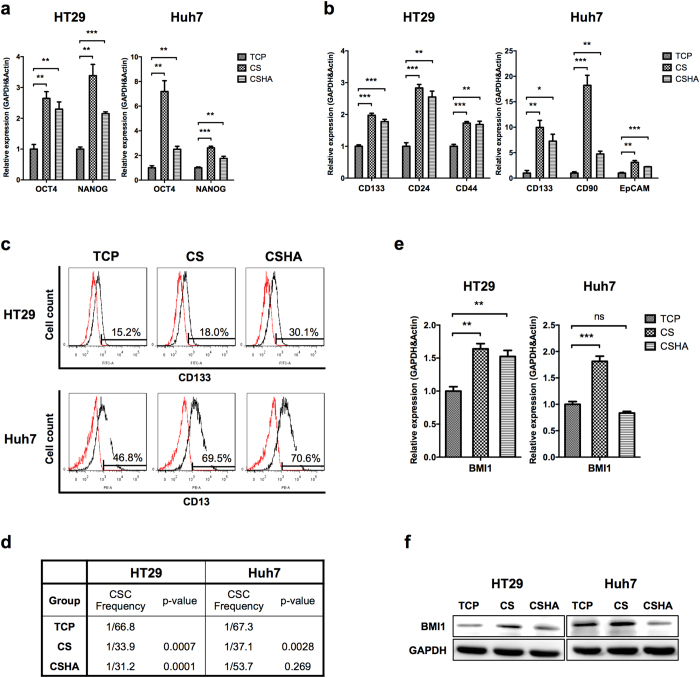
Evaluation of the stemness and the population of CSCs. (**a**) OCT4 and NANOG mRNA expressions after 72 hrs of culture. (**b**) The mRNA expression levels of CSC markers, CD133, CD24, and CD44 in colon cancer cells and CD133, CD90, and EpCAM in HCC cells after 72 hrs of culture. (**c**) The protein expression levels of CSC markers, CD133 in colon cancer cells (HT29) and CD13 in HCC cells (Huh7) by flow cytometry. (**d**) CSC frequency by ELDA sphere forming assay. (**e**) BMI1 mRNA expression after 72 hrs of culture. (**f**) The protein expression level of BMI1 after 72 hrs of culture. Each bar represents the means of three determinations ± SD. *p < 0.05, **p < 0.01, ***p < 0.001 among the indicated groups.

The stemness genes (OCT4 and NANOG) and CSC biomarkers were analyzed by RT-PCR. As shown in Fig. 4a, the expression levels of OCT4 and NANOG in both HT29 and Huh7 significantly increased on CS and CSHA membranes as compared to TCP plates. The HT29 CSC markers, CD133, CD24, and CD44, were significantly upregulated on CS and CSHA membranes (Fig. 4b). The Huh7 CSC markers, CD133, CD90, and EpCAM, were also significantly upregulated on CS and CSHA membranes (Fig. 4b). The other cell lines demonstrated similar results (Supplementary Fig. S5). The gene expression of HT29 spheres from non-adherent sphere forming culture system, another 3D culture system, was also analyzed. All CS, CSHA and sphere culture systems increased stem cell properties (Supplementary Fig. S5). Moreover, we evaluated the protein levels of CSC markers, CD133 in HT29 and CD13 in Huh7. Results showed that CD133-positive cells in HT29 increased from 15.2% on TCP plates to 18.0% and 30.1% on CS and CSHA membranes, respectively. Although the mRNA level of CD13 in Huh7 was not changed on CS and CSHA membranes as compared to TCP plates, CD13-positive cells increased from 41.1% to 66.5% and 67.6%, respectively (Fig. 4c). The ELDA sphere forming assay was performed to examine the CSC frequency and self-renewal capacity. As shown in Fig. 4d, the CSC frequencies on CS and CSHA membranes were significantly higher than those on TCP plates in both HT29 and Huh7. The expression level of BMI1, a transcription factor associated with self-renewal property, increased for HT29 on CS and CSHA membranes as compared to TCP plates. The BMI1 expression increased for Huh7 only on CS membranes (Fig. 4e,f). Besides, the side population assay was performed to evaluate the CSC ratios. Both side populations in HT29 and Huh7 cultured on CS and CSHA membranes elevated compared to TCP plates (Supplementary Fig. S4).

### CS increased the quiescent population

**Figure 5:**
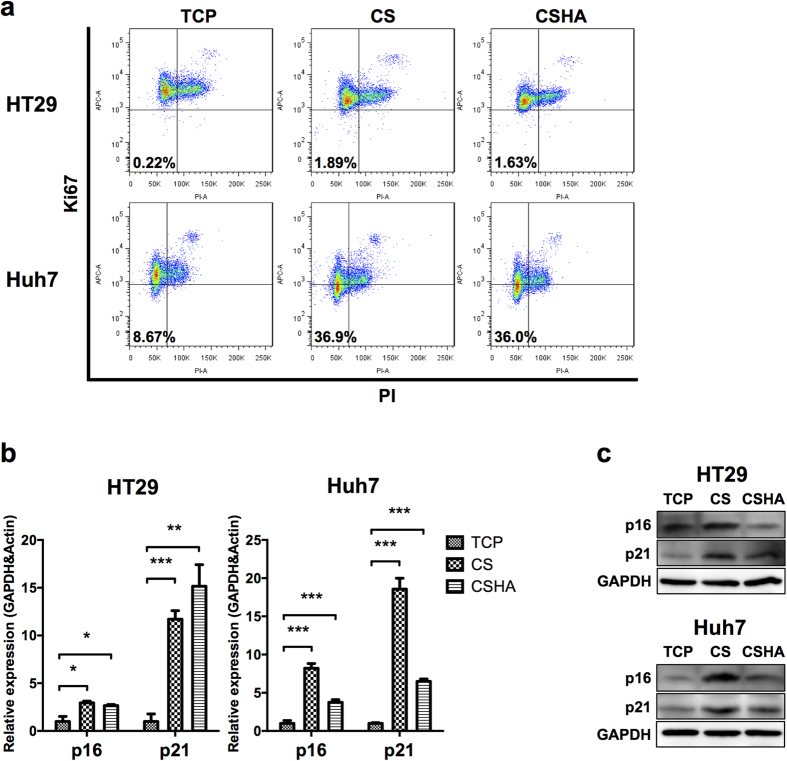
Characterization of quiescent population for HT29 and Huh7. (**a**) Ki-67 and PI negative population representing the quiescent population. (**b**) p16 and p21 mRNA expressions after 72 hrs of culture. (**c**) The protein expression levels of p16 and p21 after 72 hrs of culture. Each bar represents the means of three determinations ± SD. *p < 0.05, **p < 0.01, ***p < 0.001 among the indicated groups.

Staying at quiescent state is an important property of CSCs. The quiescent population was performed using the Ki-67/PI quiescence assay. Results revealed that the quiescent population in HT29 increased from 0.221% to 1.89% and 1.63% on CS and CSHA membranes, respectively, and that in Huh7 increased from 8.67% into 36.9% and 36.0%, respectively (Fig. 5a). The expression levels of cell cycle arrest related genes as p16 and p21 also increased on CS and CSHA membranes for both HT29 and Huh7 (Fig. 5b,c).

### Canonical and noncanonical Wnt signalings were activated in colon cancer and HCC stem cells, respectively

**Figure 6:**
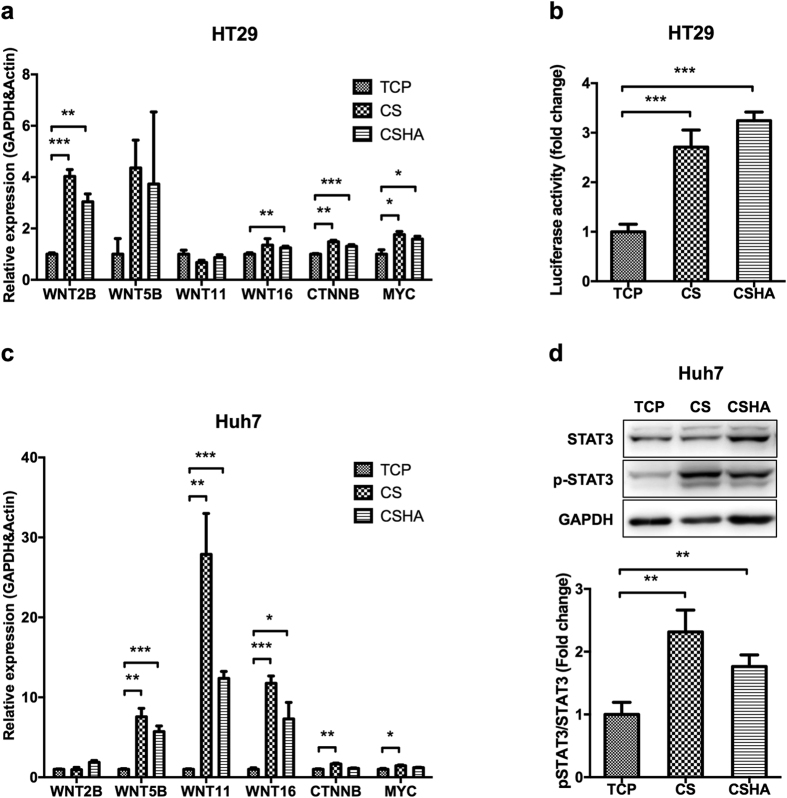
Activation of canonical and noncanonical Wnt signaling pathways for cancer cells on CS and CSHA membranes. (**a**) The mRNA expression levels of WNT2B, WNT5B, WNT11, WNT16, CTNNB, and MYC in HT29 after 72 hrs of culture. (**b**) TOP-flash luciferase reporter assay showed the activation of canonical Wnt signaling in HT29 grown on CS and CSHA membranes. (**c**) The mRNA expression levels of WNT2B, WNT5B, WNT11, WNT16, CTNNB, and MYC in Huh7 after 72 hrs of culture. (**d**) Phosphorylation of STAT3 by western blot for Huh7 on CS and CSHA membranes after 72 hrs of culture. The lower bar chart indicated the quantification of protein band. Each bar represents the means of three determinations ± SD. *p < 0.05, **p < 0.01, ***p < 0.001 among the indicated group.

The expression of canonical Wnt ligand, WNT2B, was significantly upregulated in HT29 on CS and CSHA membranes (Fig. 6a). Wnt signaling downstream genes, CTNNB and MYC, were also upregulated in HT29 on CS and CSHA membranes (Fig. 6a). The Wnt/β-catenin reporter assay (TOP-flash) showed that CS substrates activated Wnt/β-catenin signaling in HT29 but not in Huh7 (Fig. 6b, Supplementary Fig. S6). The expression of noncanonical Wnt ligands, WNT5B, WNT11, and WNT16, were upregulated in Huh7 cells on CS and CSHA membranes (Fig. 6c). Since Wnt5-Frizzled2 could activate STAT3 phosphorylation in cancer cells and increase CSC properties, we further checked the activation of STAT3. In Fig. 6d, the phosphorylated STAT3 was increased in Huh7 by culturing on CS and CSHA membranes. Meanwhile, we did not observe any change of STAT3 phosphorylation in HT29 (Supplementary Fig. S6). To confirm the role of Wnt signaling, we used IWP-4 to inhibit Wnt signaling in both HT29 and Huh7 cells (Supplementary Fig. S7). The expression levels of stemness genes as OCT4, NANOG, and CD133 decreased in the cancer cells on both CS and CSHA membranes with IWP-4 treatment. In addition, the migration ability promoted by CS and CSHA membranes was reduced with IWP-4 treatment. The cell morphology was not significantly changed between treated cells and non-treated cells (Supplementary Fig. S7).

### The CD44-dependent effect of the colon cancer cells cultured on CS and CSHA membranes

**Figure 7:**
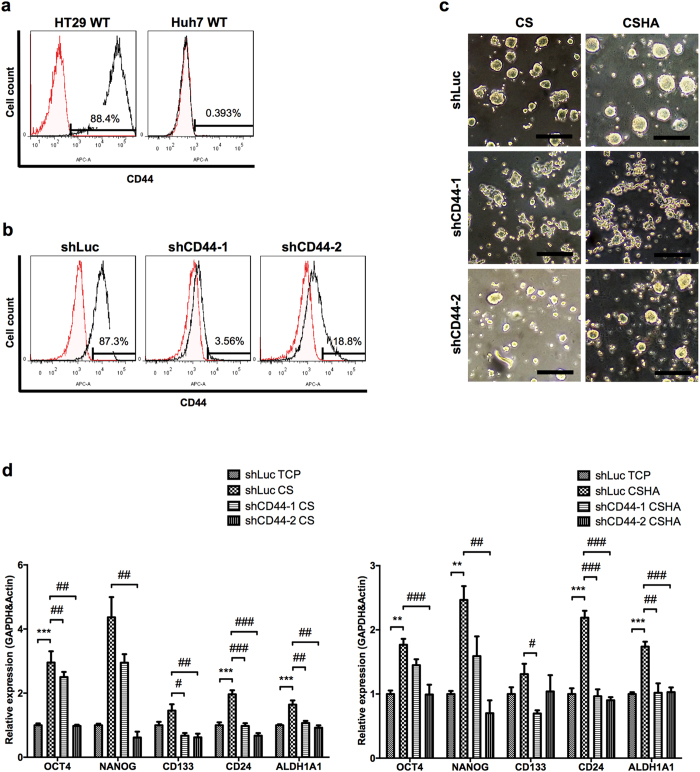
The effect of knockdown of CD44 receptor on morphology and gene expressions. (**a**) CD44 expressions in wild type (WT) HT29 and Huh7 by flow cytometry. (**b**) CD44 expressions in lentivirus-treated HT29 (shLuc, shCD44-1, and shCD44-2). (**c**) The morphology of lentivirus-treated HT29 after 72 hrs of culture. Scale bar represents 200 μm. (**d**) The mRNA expression levels of OCT4, NANOG, CD133, CD24, and ALDH1A1 for lentivirus-treated HT29 after 72 hrs of culture. Each bar represents the means of three determinations ± SD. **p < 0.01, ***p < 0.001 with respect to the shLuc TCP group. ^#^p < 0.05, ^##^p < 0.01, ^###^p < 0.001 with respect to the shLuc CS and shLuc CSHA group.

Results of cytometry revealed that HT29 had 88.4% CD44-positive cells, whereas Huh7 was a CD44-negative cell line (Fig. 7a). To investigate the role of CD44, a possible target of Wnt signaling, in the cancer cells grown on CS and CSHA membranes, knockdown of CD44 expression in HT29 was performed (Fig. 7b). The experimental results demonstrated that knockdown of CD44 significantly deprived HT29 of spheroid formation (Fig. 7c) and decreased the expression of stemness genes OCT4 and NANOG, CSC marker genes CD133 and CD24, as well as ALDH1A1 gene for HT29 both on CS and CSHA membranes (Fig. 7d).

## Discussion

Cancer cells are surrounded with stromal cells and extracellular matrix which support tumor formation and development^13^. In order to mimic *in vivo* tumor microenvironments, 3D culture with biomaterials is developed to investigate tumor biology^14^. However, scaffold matrix-embedded 3D cell culture is complicated and has difficulty in harvesting the cancer cells for further studies. Here, we used chitosan substrates to create a simple and convenient system to culture cancer cells. We showed that chitosan alone could increase cancer cell stemness properties and tumor progression.

We observed that chitosan and HA-grafted chitosan could induce diverse morphology of various cancer cell types. Cellular phenotypes, CD44 expression and cellular properties of cell lines might affect the aggregation ability on CS and CSHA. We found cells with epithelial-like phenotype and/or higher CD44 expression tended to form colonies or spheroids on CS and CSHA, in contrast to cells with mesenchymal-like phenotype and/or CD44-negative. On the other hand, CSHA provided an environment for cells to aggregate more easily than CS. Based on the cell morphology on TCP plates, HT29 and SW480 have epithelial-like phenotype, and DLD-1 and HCT116 have mesenchymal-like phenotype. As shown in our Fig. 1 and Supplementary Fig. S1, only HT29 could form spheroids on CS membranes but DLD-1 and HCT116 could not. SW480 was an exception. Previous studies^32^,^33^ and we (data not shown) revealed that SW480 could not form spheroids in the sphere forming assay. It also failed to aggregate on both CS and CSHA. HT29, DLD-1 and HCT116 are all CD44-positive cells and able to aggregate on CSHA membranes. We also observed the similar phenomenon among HCC cell lines.

Previous studies demonstrated ATP-binding cassette (ABC) transporters and aldehyde dehydrogenase (ALDH) enzymes contributed to drug resistance of cancer cells^34^,^35^. In the current study, chitosan not only elevated the cell viability of HT29 and Huh7 under 5-Fu and doxorubicin treatment but also upregulated the expression levels of ABCG2 and ALDH. Interestingly, our results showed Huh7 had higher ALDH enzyme activity while the gene expression of ALDH1A1 in Huh7 was not significantly changed on CS membranes. Probably other isoforms of ALDH contributed to the enzyme activity^36^,^37^. Besides, C-X-C chemokine receptor type 4 (CXCR4) is correlated with metastasis in colon cancer and HCC cells^38^,^39^ and activates some pathways associated with migration such as Cdc42 and Ras^40^. MMP14, a membrane-type matrix metalloproteinase (MMP), promotes cancer invasion and metastasis^41^. Our results revealed that chitosan increased the migration ability and expression levels of CXCR4 and MMP14 in both HT29 and Huh7. Knockdown of MMP14 could reduce the effect of chitosan substrates on cell motility in both cell lines. However, knockdown of CXCR4 by several shRNA sequences in Huh7 could not significantly decrease the migration ability in CS and CSHA groups. We assumed that dozens of genes and cellular properties might be changed while cells grew on CS or CSHA. Several motility-related genes might be upregulated to enhance the cellular migration ability by CS and CSHA. Therefore, although CXCR4 plays an important role in the migration of Huh7 cells^42^, knockdown of CXCR4 gene might not significantly affect the migration of Huh7 cells in CS and CSHA groups. According to GSEA analysis, we also found genes related to Cdc42 and Ras pathways were upregulated on chitosan membranes. In addition, mTOR, a downstream effector of the PI3K/AKT pathway has been reported to affect tumor progression^43^. By means of the GSEA analysis, we found that the mTOR pathway associated genes were upregulated for HT29 cells on chitosan membranes. GSEA data also revealed that the upregulated genes were associated with the EGFR or Myc target gene set. Several studies have shown that EGFR and Myc are often overexpressed in human carcinomas and play crucial roles in tumorigenesis^44^,^45^. On the other hand, we observed both CS and CSHA had similar stemness-induced effects with varying degrees on the cancer cells. CSHA was a biomaterial with grafted HA on the surface of CS. Therefore, HA was the major component of CSHA to interact with the cancer cells. Even though previous study suggested the structure of HA is partially similar to chitosan^30^, CS and CSHA might create similar but different microenvironments to affect the cancer cells. Taken together, chitosan alone may enhance the cancer cell migration ability and drug resistance and may activate several tumorigenesis-associated pathways.

Recent literature showed that CSCs, distinct subpopulation cells in the tumor, contributed to cancer heterogeneity that increased the complexity within a tumor and caused therapeutic problems^3^. However, CSCs would decrease stemness properties and differentiate into the progeny cancer cells when they lost their specific microenvironments^19^,^20^. Our results showed that the chitosan culture system could mimic tumor microenvironments and elevate the expression levels of stemness and CSC marker genes. The heatmap analysis of microarray data demonstrated that the upregulated genes of HT29 cultured on chitosan substrates were correlated with the stemness gene set of normal Lgr5-positive intestinal stem cells^46^. CSC marker genes of HT29 cells were also upregulated on chitosan membranes. These data revealed that chitosan might increase stemness-related genes. We further verified these data by RT-PCR analyses and CSC-associated functional assays. We found that the core stemness transcription factors, OCT4 and NANOG, increased significantly for both HT29 and Huh7. Besides, the expression levels of CSC markers, CD133, CD24, CD44, CD90, and EpCAM were enhanced. These CSC markers are also required for maintenance of stemness properties of CSCs through several signaling pathways^47^,^48^. The factors associated with drug resistance, ABCG2, and ALDH, were also regarded as the CSC markers^49^,^50^. Moreover, CSCs maintain CSC pools through their self-renewal ability that could be evaluated by sphere forming assay^51^. The results of ELDA sphere forming assay revealed that the cancer cells on the chitosan membranes increased not only self-renewal ability but also the CSC frequency. Chitosan also increased the BMI1 expression, the crucial regulator of self-renewal^52^. Additionally, some CSCs in the tumors stay at quiescent states, regarded as G_0_ phase in cell cycles, to prevent CSC exhaustions caused by differentiation^53^. At quiescent states, CSCs could resist anti-proliferation drugs and then escape from cytotoxic chemotherapies^54^. Our results revealed that chitosan increased quiescent population and the expression of quiescence regulators p16 and p21 in both HT29 and Huh7. These results suggested chitosan might increase CSC population via increasing quiescence of CSCs. Taken together, we demonstrated that chitosan substrates promoted the characteristics of CSCs such as migration ability, drug resistance, self-renewal capacity, and quiescent population.

A few biomaterials were used in cancer cell culture systems to imitate the tumor microenvironments^14^. These biomaterial scaffolds facilitated cancer cells to form 3D multicellular spheroids that mimic the solid tumor *in vivo*. Other reports showed that formation of such 3D cell spheroids could increase the stemness of cancer cells^55^,^56^. In the present study, chitosan promoted the stemness properties of cancer cells regardless if cells were assembled into 3D cellular spheroids. Chitosan itself may provide external signals from microenvironments to alter the fate of cancer cells, but the interactions between chitosan and cancer cells were relatively unexplored. Multiple signaling pathways including Wnt/β-catenin, Notch, and Hedgehog regulate the CSC properties^8^. Colon CSC population possesses high Wnt activity^9^. Hyperactivation of Wnt/β-catenin signaling could even trigger differentiated epithelial cells to convert into tumor-initiating cells^57^. Noncanonical Wnt signaling also enhances CSC characteristics in HCC and colon cancers^10^. Noncanonical Wnt ligand can increase EMT in HCC cells through STAT3 activation. Here, we showed that chitosan significantly increased the phosphorylation of STAT3 in Huh7 HCC cells but not in HT29 colon cancer cells. Nevertheless, we observed chitosan increased the Wnt/β-catenin reporter activity in HT29 cells. Thus, we suggested that the chitosan culture system increased canonical and noncanonical Wnt signalings in HT29 and Huh7 cancer cells, respectively. On the other hand, Rao *et al*. recently revealed that chitosan nanoparticles could bind to cell membranes of CSCs via CD44^30^. The chemical structure of chitosan partially resembles HA, a major ligand of CD44 receptors. Thus, they assumed that chitosan may specifically target CD44^positive^ CSCs. Additionally, CD44 isoforms on CSCs could couple with other signaling pathways, such as c-Met, which could promote their stemness properties^58^. CD44 is a Wnt/β-catenin target gene and can also activate Wnt/β-catenin in a concentration-dependent manner via modulating Wnt-LRP6 receptor complex^59^. Our results demonstrated that knockdown of the CD44 expression in HT29 would abolish their ability to form multicellular spheroids on chitosan membranes and downregulate the stemness and CSC marker genes. Taken together, chitosan substrates increase CSC properties of HT29 colon cancer cells and Huh7 HCC cells and probably are associated with different Wnt signaling pathways.

**Figure 8:**
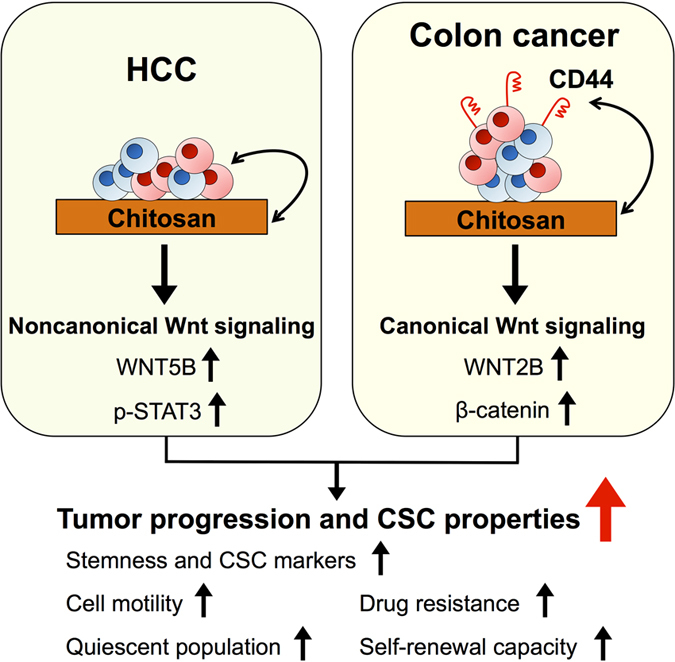
Chitosan might promote tumor progression and CSC properties in association with canonical or noncanonical Wnt signalings in different cancer cells.

Chitosan modified with other molecules has been recently used as culture systems for the investigation of tumor progressions and has been shown to increase the cancer stemness^23^,^24^,^25^,^26^. In the current study, we considered that the pristine chitosan per se could promote the properties of CSCs including migration ability, drug resistance, quiescent population, and self-renewal capacity through in colon cancers. We also verified that chitosan substrates activated canonical and noncanonical Wnt signaling pathways in CD44^positive^ colon cancer and CD44^negative^ HCC, respectively (Fig. 8). This simple culture system mimics *in vivo* microenvironments of CSCs and creates a novel platform for CSC research and cancer drug development.

## Materials and Methods

### Chitosan (CS) and HA-grafted chitosan (CSHA) membranes preparation

The molecular weight of the chitosan powder (Sigma) was 510 kDa and the degree of deacetylation was 77%. The molecular weight of HA (SciVision Biotech) was about 2500 kDa. CS and CSHA membranes were prepared as previously reported^29^. Briefly, chitosan was dissolved in 1% acetic acid solution and coated on each well of 6-well plate. CS membranes were formed after evaporation of solvent in a laminar flow cabinet for 24 hrs. CS membranes were soaked in 0.5 N sodium hydroxide for 30 minutes, and washed with phosphate buffered saline (PBS) three times. For the preparation of CSHA membranes, HA solution was added in each chitosan-coated well with the amount of 0.5 mg per cm^2^ of CS membranes. CSHA membranes were crosslinked with ethyl (dimethylaminopropyl) carbodiimide/N-hydroxysuccinimide (EDC/NHS) solution with a weight ratio of HA/EDC/NHS adjusted to 1:1.84:0.23 at pH 5.5. Then, CSHA membranes were washed with PBS three times to remove unbound HA.

### Cell culture and cell seeding on CS and CSHA membranes

The human colon cancer cell lines HT29 (ATCC) and DLD-1 (ATCC) were cultured with Roswell Park Memorial Institute (RPMI, Life Technologies) 1640 medium. The human colon cancer cell lines, HCT116 (ATCC) and SW480 (ATCC), and HCC cell lines Huh7 (JCRB), HepG2 (ATCC), Hep3B (ATCC), and SKHep-1 (ATCC) were cultured with Dulbecco’s modified Eagle’s medium (DMEM, Life Technologies). Both RPMI and DMEM medium contained 10% fetal bovine serum (FBS, GeneDireX) and 1% penicillin, streptomycin, and glutamine (PSG, Life Technologies). Cells were maintained at 37 °C in a humidified atmosphere of 5% CO_2_ within 10 passages. Cells were seeded on CS and CSHA membranes or tissue culture polystyrene (TCP) plates as the control group. The microscopy and real-time Cultured Cell Monitoring System were used for the observation of cell morphology. For Wnt signaling inhibition experiment, cells were treated with IWP-4 (20 μM, STEMCELL Technologies) for 24 hrs after seeded on CS and CSHA membranes.

### Transwell migration assay

Culture wells were divided into upper and lower compartments with transwell inserts (8 μm pore size, BD Bioscience). After starvation with serum-free medium, cells (2 × 10^5^ cells/well) were harvested and plated into the upper compartment which contained 300 μL serum-free medium, while the lower compartment contained 500 μL medium with 10% FBS. Following 18 hrs of incubation at 37 °C in 5% CO_2_, non-migrated cells were removed from the upper surface of the transwell, and the migrated cells remaining on the lower surface were fixed with methanol for 10 min and counted after staining with crystal violet for 1 hr.

### Drug resistance assay

While seeding on CS and CSHA membranes or TCP plates, HT29 and Huh7 were treated with varying concentrations of 5-Fluorouracil (Sigma) and doxorubicin (Sigma) for 72 hrs, respectively. Cell viability was evaluated using MTT (Sigma) assay according to the manufacturer’s instructions.

### Flow cytometry analysis

HT29 and Huh7 were stained with FITC-conjugated CD133 (Miltenyi Biotec) and PE-conjugated CD13 (BD Bioscience) at 4 °C for 1 hr, respectively. Cell staining with FITC-conjugated IgG (Biolegend) or PE-conjugated IgG (Biolegend) was used as the isotype control group. Aldehyde dehydrogenase (ALDH) activity was measured using ALDEFLUOR reagent (STEMCELL Technologies) with or without DEAB (ALDH inhibitor) following the manufacturer’s instructions. For analysis of side population, cells were incubated in 10% FBS medium containing 5 μg/mL Hoechst 33342 (Life Technologies) at 37 °C for 90 minutes. In control groups, cells were incubated with Hoechst 33342 in the presence of 50 μM Verapamil (Sigma). For quiescence assay, the quiescence (G_0_ phase) was defined as the population of Ki-67 negative cells in G_0_/G_1_ phase divided with propidium iodide (PI). In the assay, Foxp3/Transcription Factor Staining Buffer Set (eBioscience) was used for fixation and permeabilization following the manufacturer’s instructions. Cells were incubated with anti-human Ki-67 biotin (eBioscience) at room temperature for 1 hr. Then, cells were incubated with APC-conjugated streptavidin (Biolegend), 1 mg/mL RNase A (Sigma) and 30 μg/mL PI (Life Technologies) at room temperature for 30 minutes. After the incubation, all samples were resuspended, passed through cell strainers (40 μm, BD Bioscience), and analyzed by flow cytometer (BD FACSCanto II and BD LSRFortessa) or cell sorter (BD FACSAria III).

### Microarray data analysis

Total RNA was prepared using TriPure Isolation Reagent (Roche Life Science). cRNA was amplified and labelled using a Quick Amp Labelling Kit (Agilent Technologies) and hybridized to a 8 × 60 k v2 array chips (Agilent Technologies) according to the manufacturer’s instructions. The hybridized microarray slides were scanned using an Agilent scanner. The relative hybridization intensities and background hybridization values were calculated using Feature Extraction Software version 9.5.1.1 (Agilent Technologies). The raw signal intensities and flags for each probe were calculated from the hybridization intensities and spot information according to the procedures recommended by Agilent Technologies. Briefly, a signal intensity less than 1 was corrected to 1 (not detected), and then the 75th percent shift normalization was conducted. Unless otherwise specified, data processing and analysis were performed using the statistical software R. Gene Set Enrichment Analysis (GSEA)^60^ was performed according to the standard procedure. Stemness gene set was adopted from the publication by Munoz, J *et al*.^46^.

### Sphere forming assay and 3D culture system

ELDA (extreme limiting dilution analysis) was performed to evaluate self-renewal capacity^61^. Briefly, cells were dissociated into single cell suspensions and plated at the concentration of 100, 50 and 25 cells per well, in Ultra-Low attachment 96-well plate (Corning) with serum-free sphere medium containing 2% B27 supplement (Gibco), 20 ng/mL epidermal growth factor (EGF) (PeproTech), and 10 ng/mL basic fibroblast growth factor (bFGF) (PeproTech). After 10 days of culture, spheres were stained by Hoechst 33342 (Life Technologies) and counted by IN Cell Analyzer 2000 (GE Healthcare Life Sciences). The frequency of sphere-forming cells was determined using the ELDA web-based tool at http://bioinf.wehi.edu.au/software/elda. Non-adherent and long-term culture was used for 3D sphere culture system. Briefly, cells were seeded into the agarose-coated plate with serum-free sphere medium containing 2% B27 supplement (Gibco), 20 ng/mL EGF (PeproTech), 10 ng/mL bFGF (PeproTech). After 9 days culture, spheres were harvested and analyzed by RT-PCR.

### Luciferase reporter assay

The TOP-flash luciferase assay is widely used to measure Wnt signaling^62^. The TOP-flash luciferase reporter construct contains 3 copies of the Tcf/LEF-binding site (AAGATCAAAGGGGGT) upstream of a TK minimal promoter. Cells were transfected with TOP-flash and pmCherry-C1 (Clontech) plasmid by JetPRIME reagent (Polyplus-transfection) following the manufacturer’s protocol. After 24 hrs of incubation, cells were harvested and seeded on TCP plates, CS and CSHA membranes for 48 hrs of culture. For luminescence measurement, cell lysates were transferred to white 96-well microplate and then the measurement was performed using Orion L Microplate Luminometer (Berthold Detection Systems). Fluorescence intensity of mCherry was measured by FlexStation 3^®^ microplate reader (Molecular Devices) for internal control.

### Western blot

Cells were lysed in cold radioimmunoprecipitation assay (RIPA) lysis buffer containing protease inhibitor (Sigma) and phosphatase inhibitor (Sigma) and subjected to sodium dodecyl sulfate polyacrylamide gel electrophoresis (SDS-PAGE). Proteins were transferred onto polyvinylidene difluoride (PVDF) membranes and probed with primary antibodies. The protein expression levels were normalized with GAPDH (Millipore). The information of the antibodies was shown in Supplementary Table S2.

### Lentivirus knockdown system

The plasmids of short hairpin RNA (shRNA) targeting CD44, MMP14, and CXCR4 were purchased from Academia Sinica, Taipei, Taiwan. To prepare shRNA lentiviral stocks, HEK293T cells were transfected with three lentivirus-packaging plasmids (pMDLg/pRPE, pMD2G, pRSV-Rev, Addgene) and CD44 shRNA (shCD44-1 or shCD44-2), MMP14 shRNA (shMMP14-1 or shMMP14-2), CXCR4 shRNA (shCXCR4-1 or shCXCR4-2), or luciferase shRNA (shLuc) plasmids by JetPRIME (Polyplus-transfection) reagent following the manufacturer’s protocol. After 48 hrs of incubation, supernatants containing shRNA lentivirus were collected by centrifugation with 8.5% polyethylene glycol 6000 and 0.3 M sodium chloride. Then, lentivirus was introduced into HT29 or Huh7 for 24 hrs, followed by selection with puromycin (1 μg/mL) for 48 hrs. Flow cytometry and western blot were used to confirm the knockdown efficiency.

### RNA extraction and real-time polymerase chain reaction (RT-PCR)

RNA was extracted using the TriPure Isolation Reagent (Roche Life Science). cDNA was prepared using the High-Capacity cDNA Reverse Transcription Kit (Applied biosystem). RT-PCR was performed using iQ SYBR green detection system in CFX96 (Bio-Rad). The expression level of target genes was normalized to GAPDH and β-actin. The primers used for RT-PCR were shown in Supplementary Table S1.

### Statistical analysis

Data were expressed as means ± SD. Statistical significance of results was evaluated using ANOVA and further analyzed by Tukey’s multiple comparison test. All statistical tests were performed using GraphPad Prism v.5.0. P-value were displayed on the graphs using a single asterisk for values between 0.05 to 0.01, two asterisk for values between 0.01 to 0.001, and three asterisks for values below 0.001.

## Additional Information

**How to cite this article**: Chang, P.-H. *et al*. Chitosan promotes cancer progression and stem cell properties in association with Wnt signaling in colon and hepatocellular carcinoma cells. *Sci. Rep.* **7**, 45751; doi: 10.1038/srep45751 (2017).

**Publisher's note:** Springer Nature remains neutral with regard to jurisdictional claims in published maps and institutional affiliations.

## Supplementary Material

Supplementary Information

Supplementary Video 1

Supplementary Video 2
