# Supplementary material for: Chitosan promotes cancer progression and stem cell properties in association with Wnt signaling in colon and hepatocellular carcinoma cells
Source: Sci Rep. 2017 Apr 3;7:45751. doi: 10.1038/srep45751 (PMC5377948; doi:10.1038/srep45751)
Supplement: Supplementary Information [file srep45751-s1.pdf]

Supplementary Information

Chitosan promotes cancer progression and stem cell properties in association with Wnt signaling in colon and hepatocellular carcinoma cells

Po-Hsiang Chang[1], Keisuke Sekine[2], Hsiao-Mei Chao[1, 3], Shan-hui Hsu[4, *], Edward Chern[1, *]

[1]niChe Lab for Stem Cell and Regenerative Medicine, Department of Biochemical Science and Technology, National Taiwan University, Taipei 10617, Taiwan

[2]Department of Regenerative Medicine, Yokohama City University Graduate School of Medicine, Kanagawa 236-0004, Japan

[3]Department of Pathology, Wan Fang Hospital, Taipei Medical University, Taipei 11696, Taiwan

[4]Institute of Polymer Science and Engineering, National Taiwan University, Taipei 10617, Taiwan

*Corresponding authors. Email: shhsu@ntu.edu.tw or edchern@ntu.edu.tw

Supplementary video

The video clips of HT29 colon cancer cells and Huh7 HCC cells grown on chitosan membranes showed the dynamic processes of spheroids and colonies formation. Both videos were acquired from 0 to 72 hrs after cell seeding.

#1. HT29 cells seed on CS membranes.

#2. Huh7 cells seed on CS membranes.

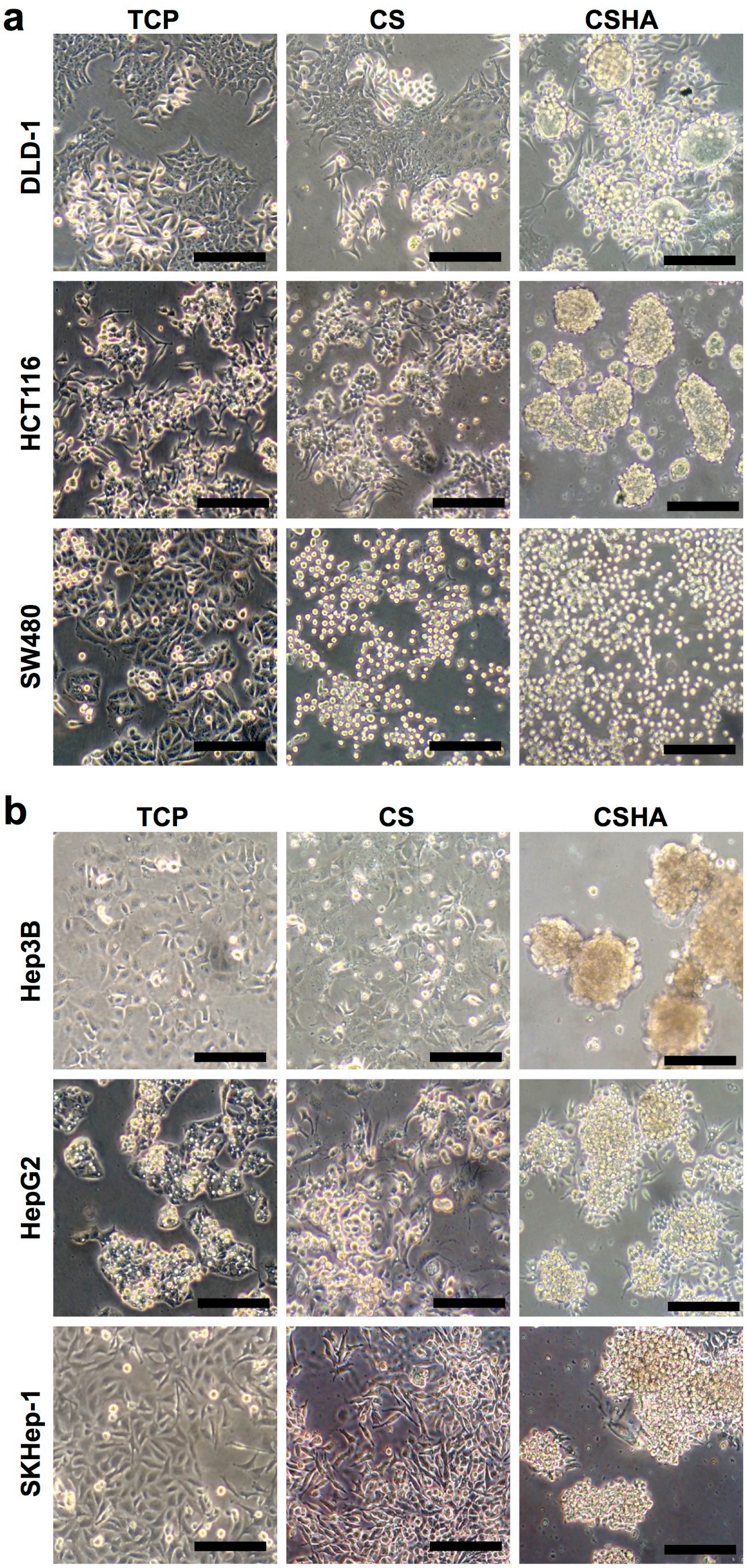

Figure S1. Morphology of some other cancer cell lines grown on CS or CSHA membranes. (a) Colon cancer cell lines, DLD-1, HCT116, and SW480 cultured for 72 hrs. (b) HCC cell lines, Hep3B, HepG2, and SKHep-1 cultured for 72 hrs. Scale bar represents 200 μm.

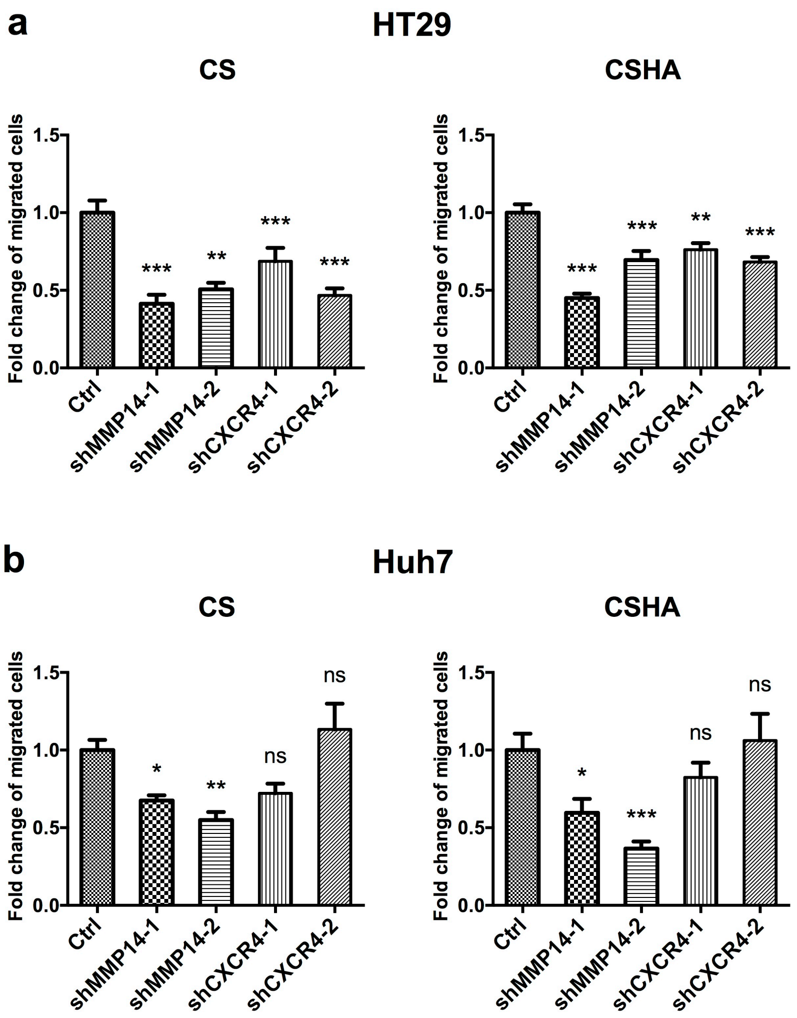

Figure S2. The effect of MMP14 or CXCR4 knockdown on migration ability. (a) The normalized migrated HT29 cells in CS and CSHA groups. (b) The normalized migrated Huh7 cells in CS and CSHA groups. Each bar represents the means of three determinations ±SD. *p < 0.05, **p < 0.01, ***p < 0.001 among the control groups.

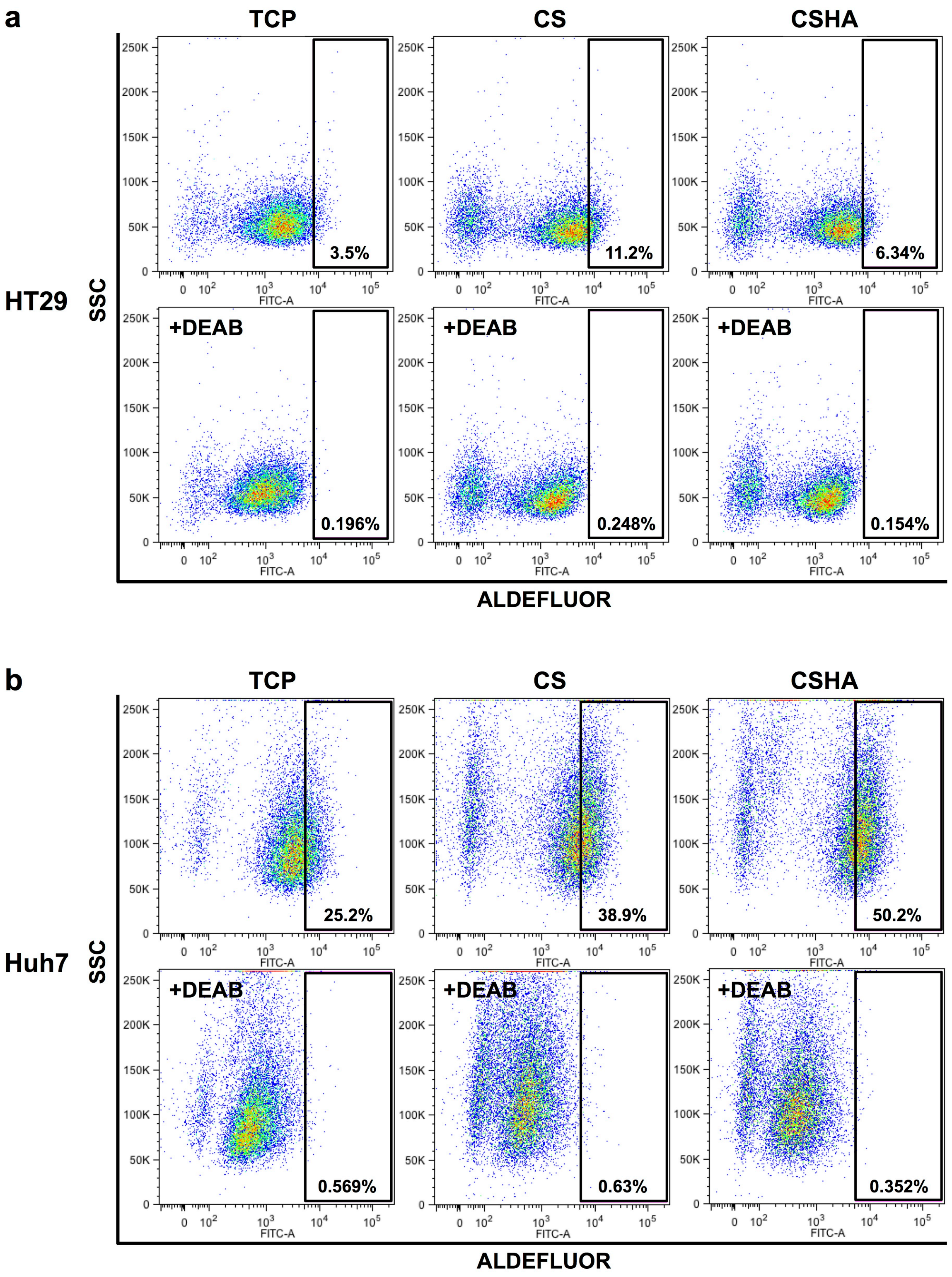

Figure S3. Evaluation of ALDH activity by ALDEFLUOR assay. (a) The ALDH-positive cells in HT29 increased from 3.5% on TCP plates to 11.2% and 6.34% on CS and CSHA membranes, respectively. (b) The ALDH-positive cells in Huh7 increased from 25.2% on TCP plates to 38.9% and 50.2% on CS and CSHA membranes, respectively.

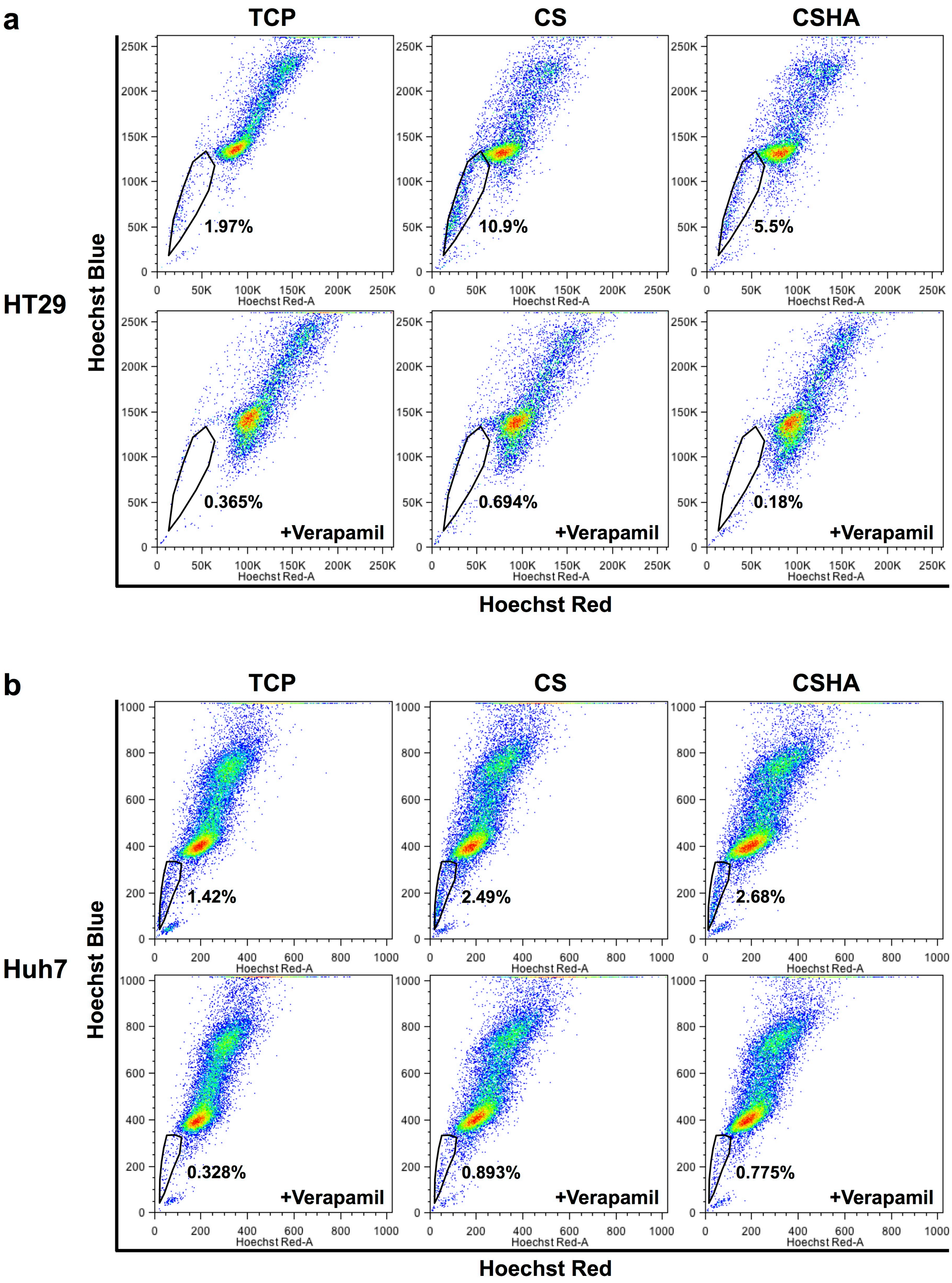

Figure S4. Characterization of ABC transporter function and CSC ratio via side population (SP) assay. (a) The SP-positive cells in HT29 increased from 1.97% on TCP plates to 10.9% and 5.5% on CS and CSHA membranes, respectively. (b) The SP-positive cells in Huh7 increased from 1.42% on TCP plates to 2.49% and 2.68%, respectively.

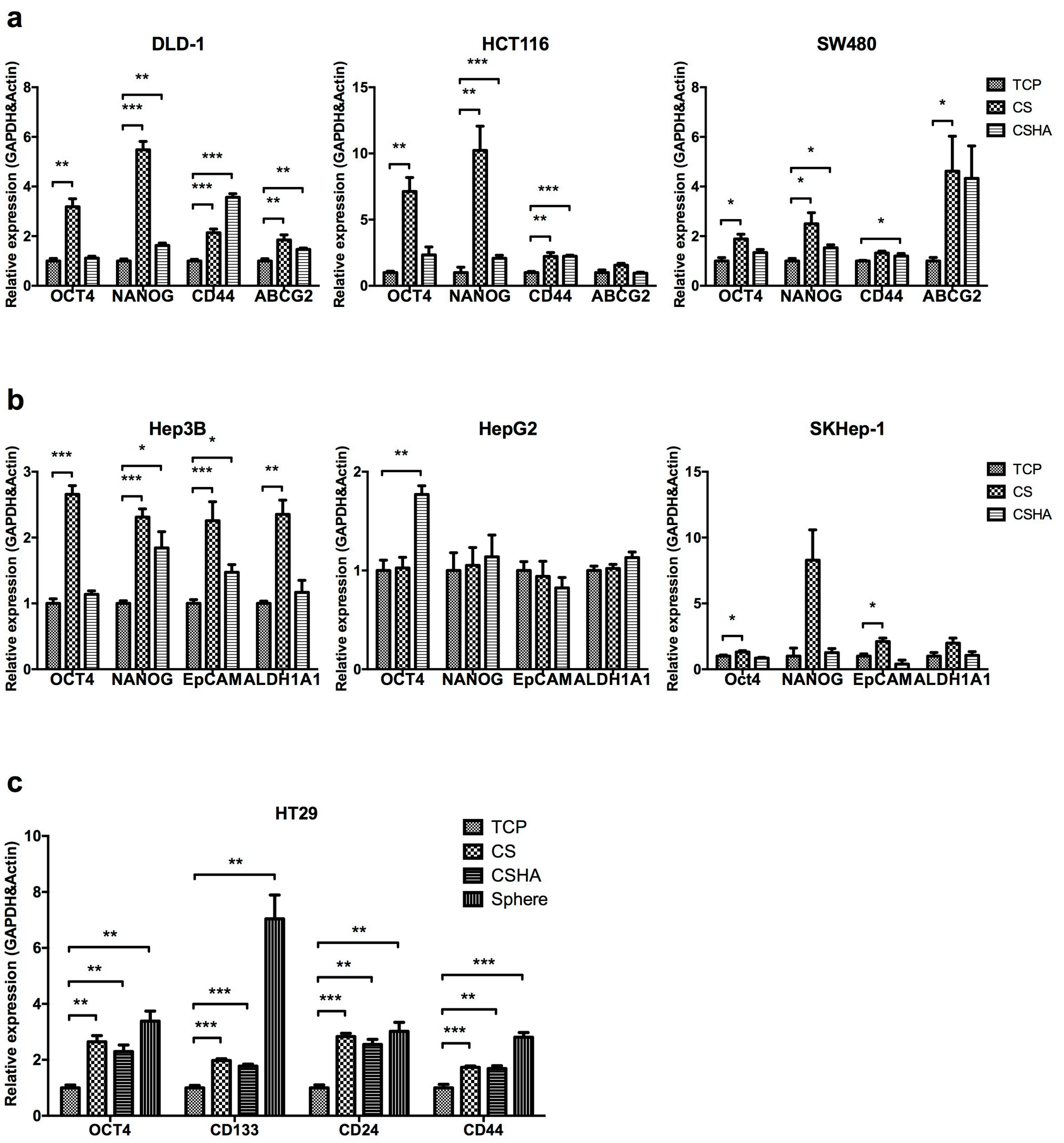

Figure S5. Analysis of expression levels of stemness (OCT4 and NANOG), CSC markers (CD133, CD24, and CD44 in colon cancer and EpCAM in HCC) and chemoresistance related gene (ABCG2 and ALDH1A1) in some other cancer cell lines and 3D culture system. (a) Colon cancer cell lines, DLD-1, HCT116, and SW480. (b) HCC cell lines, Hep3B, HepG2, and SKHep-1. (c) Comparison of TCP, CS, CSHA system and 3D sphere culture system. Each bar represents the means of three determinations ±SD. *p < 0.05, **p < 0.01, ***p < 0.001 among the indicated groups.

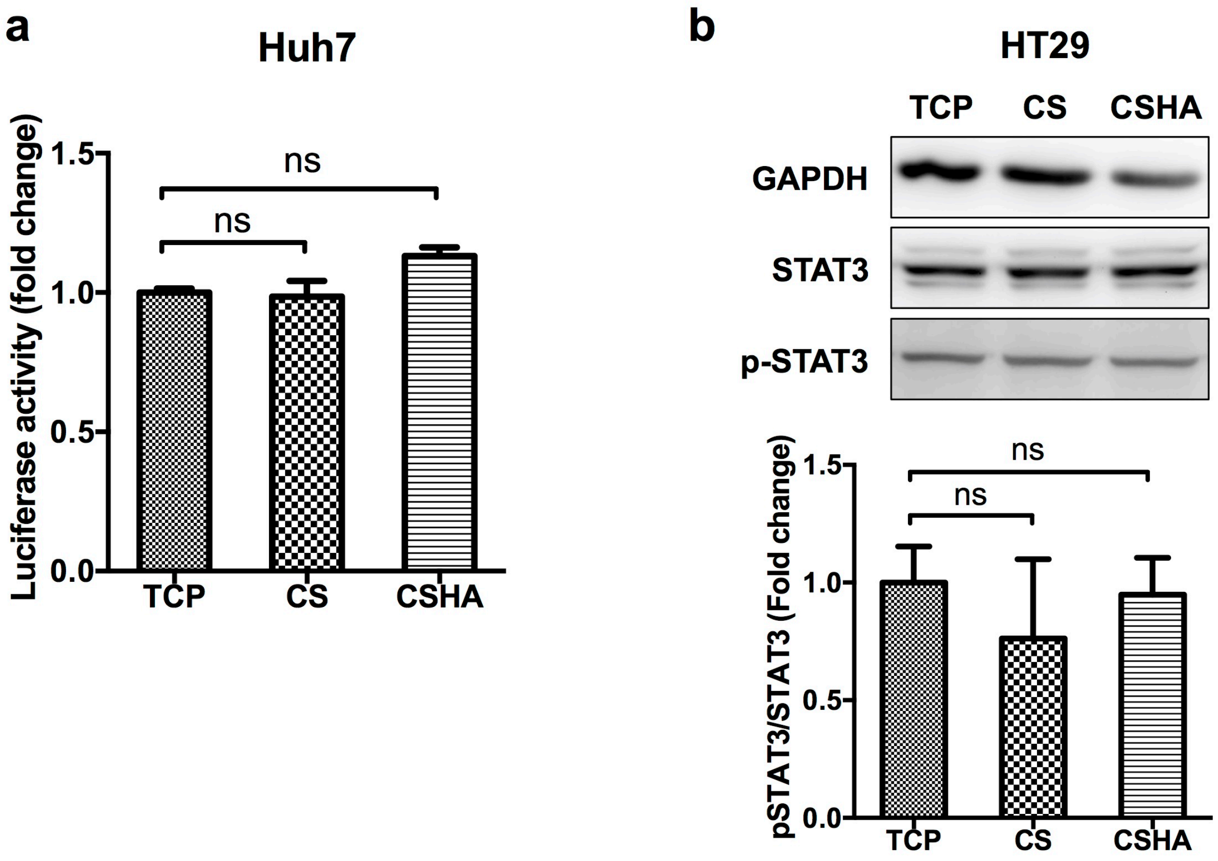

Figure S6. Canonical and noncanonical Wnt signalings were measured by the Wnt/β-catenin reporter assay and STAT3 phosphorylation, respectively. (a) Compared to TCP plates, the luciferase activity in Huh7 demonstrated no significant difference for cells on CS or CSHA membranes. (b) The STAT3 phosphorylation in HT29 revealed no significant difference among TCP plates, CS, and CSHA membranes. Each bar represents the means of three determinations ±SD.

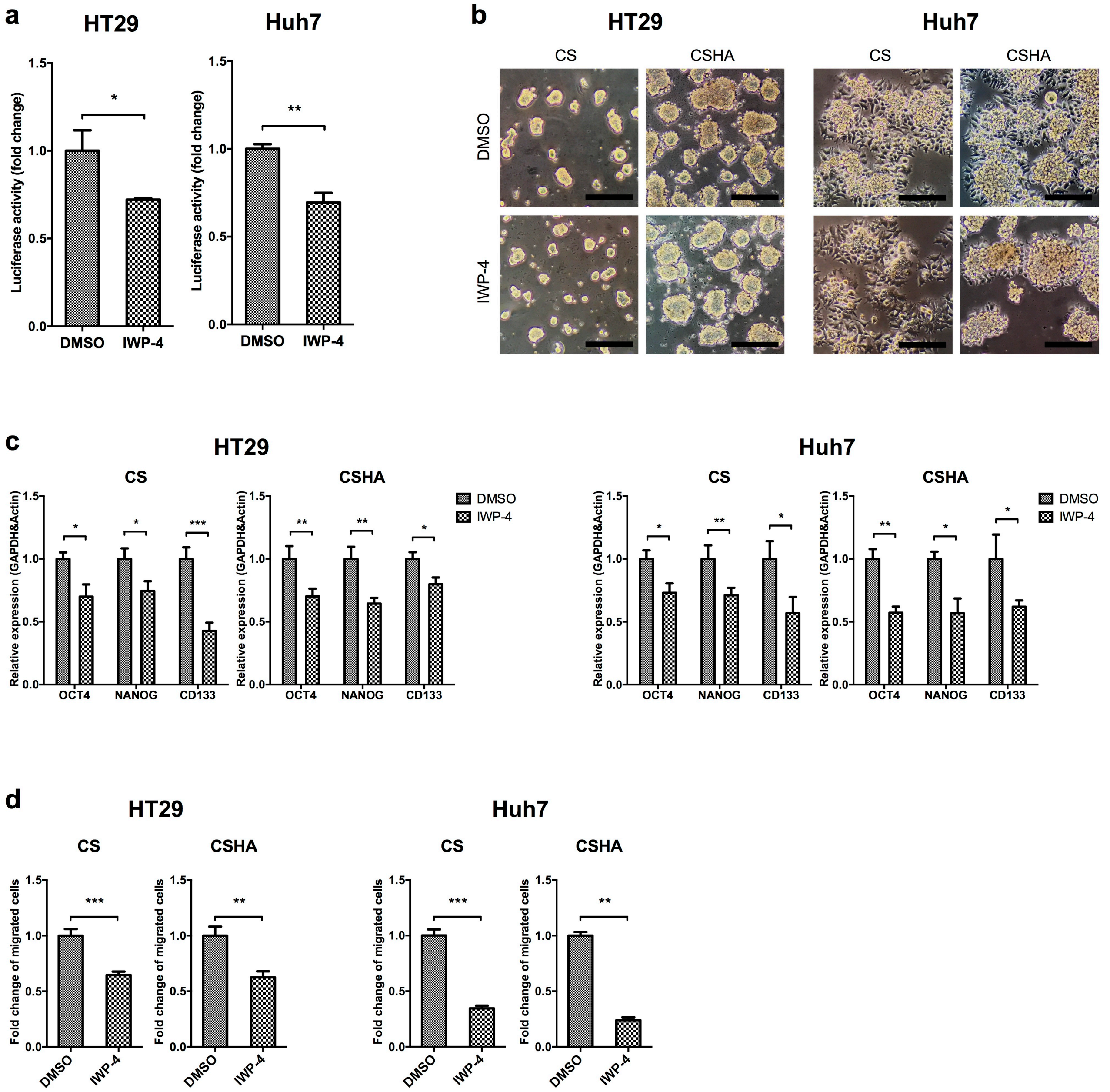

Figure S7. The effect of Wnt signaling inhibition with IWP-4 treatment on morphology and gene expressions. (a) The luciferase reporter assay demonstrated the inhibition efficacy. (b) The morphology of HT29 and Huh7 grown on CS and CSHA membranes. Scale bar represents 200 μm. (c) The expression levels of OCT4, NANOG, and CD133 after 72 hrs of culture. (d) The normalized migrated HT29 and Huh7 cells in CS and CSHA groups. Each bar represents the means of three determinations ±SD. *p < 0.05, **p < 0.01, ***p < 0.001 among the indicated groups.

Table S1. The information of RT-PCR primers and shRNA.

Gene	Primer sequences
CXCR4	F: ACTACACCGAGGAAATGGGCT<br>R: CCCACAATGCCAGTTAAGAAGA
MMP14	F: CGAGGTGCCCTATGCCTAC<br>R: CTCGGCAGAGTCAAAGTGG
ABCG2	F: ACGAACGGATTAACAGGGTCA<br>R: CTCCAGACACACCACGGAT
ALDH1A1	F: CTGCTGGCGACAATGGAGT<br>R: CGCAATGTTTTGATGCAGCCT
OCT4	F: GACAGGGGGAGGGGAGGAGCTAGG<br>R: CTTCCCTCCAACCAGTTGCCCCAAAC
NANOG	F: TTTGTGGGCCTGAAGAAAACT<br>R: AGGGCTGTCCTGAATAAGCAG
CD133	F: ACAGCATATTCTTCACGCAGAGAT<br>R: GTCAGATGGAGTTACGCAGGTT
CD24	F: CTCCTACCCACGCAGATTTATTC<br>R: AGAGTGAGACCACGAAGAGAC
CD44	F: GCAGTCAACAGTCGAAGAAGG<br>R: TGTCCTCCACAGCTCCATT
CD90	F: GCTTATGGCATCTCATTGAGGACA<br>R: CAGGTGGACACGAGGACAGA
EpCAM	F: ATAATCGTCAATGCCAGTGTA<br>R: TTTGCTCTTCTCCCAAGTTT
p16	F: GATCCAGGTGGGTAGAAGGTC<br>R: CCCCTGCAAACTTCGTCCT
p21	F: TGTCCGTCAGAACCCATGC<br>R: AAAGTCGAAGTTCCATCGCTC
BMI1	F: TGGACTGACAAATGCTGGAGA<br>R: GAAGATTGGTGGTTACCGCTG
WNT2B	F: CGGGACCACACCGTCTTTG<br>R: GCGAGTAATAGCGTGGACTAC
WNT5B	F: CATGGCCTACATAGGGGAGG<br>R: CTGTGCTGCAATTCCACCG
WNT11	F: GGAGTCGGCCTTCGTGTATG<br>R: GCCCGTAGCTGAGGTTGTC
WNT16	F: AGTATGGCATGTGGTTCAGCA<br>R: GCGGCAGTCTACTGACATCAA

CTNNB	F: CATCTACACAGTTTGATGCTGCT<br>R: GCAGTTTTGTCAGTTCAGGGA
MYC	F: GTCAAGAGGCGAACACACAAC<br>R: TTGGACGGACAGGATGTATGC
GAPDH	F: GGAGCGAGATCCCTCCAAAAT<br>R: GGCTGTTGTCATACTTCTCATGG
β-actin	F: GGCACCCAGCACAATGAAG<br>R: CCGATCCACACGGAGTACTTG

shRNA	Oligonucleotide sequences
MMP14-1	CCGGGCTGAGATCAAGGCCAATGTTCTCGAGAACATTGGCCTT<br>GATCTCAGCTTTTTG
MMP14-2	CCGGGTGGTGTTCCAGACAAGTTTGCTCGAGCAAACTTGTCTG<br>GAACACCACTTTTTTG
CXCR4-1	CCGGCCTGTTCTTAAGACGTGATTTCTCGAGAAATCACGTCTTA<br>AGAACAGGTTTTTG
CXCR4-2	CCGGTCCTGTCCTGCTATTGCATTACTCGAGTAATGCAATAGCA<br>GGACAGGATTTTTG
CD44-1	CCGGCCGTTGGAAACATAACCATTACTCGAGTAATGGTTATGT<br>TTCCAACGGTTTTTG
CD44-2	CCGGGGACCAATTACCATAACTATTCTCGAGAATAGTTATGGT<br>AATTGGTCCTTTTTG

Table S2. The information of antibodies.

Antibody	Manufacturer	Catalog Number	Application	Dilution Fold or Concentration
Primary antibodies				
Anti-CD133	Miltenyi Biotec	130-105-225	Flow cytometry	1/11
Anti-CD13	BD Bioscience	560998	Flow cytometry	1/200
Anti-Ki67	eBioscience	13-5699-82	Flow cytometry	1/400
Mouse isotype IgG	Biolegend	400107/400113	Flow cytometry	1/200
Anti-CXCR4	Abcam	ab124824	Western blot	1/500
Anti-MMP14	Abcam	ab51074	Western blot	1/2000
Anti-BMI1	Abacm	ab14389	Western blot	2 μg/mL
Anti-p16	VENTANA	705-4713	Western blot	1/100
Anti-p21	Cell Signaling Technology	2947S	Western blot	1/1000
Anti-STAT3	Santa Cruz	sc-482	Western blot	1/2000
Anti-p-STAT3	Abcam	ab76315	Western blot	1/2000
Anti-GAPDH	Millipore	MAB374	Western blot	1/10000
Secondary antibodies				
APC streptavidin	Biolegend	405207	Flow cytometry	1/400
Anti-mouse HRP	Jackson ImmunoResearch	115-035-003	Western blot	1/10000
Anti-rabbit HRP	Jackson ImmunoResearch	111-035-003	Western blot	1/10000
